# TRIM8 inhibits porcine epidemic diarrhoea virus replication by targeting and ubiquitinately degrading the nucleocapsid protein

**DOI:** 10.1186/s13567-024-01443-2

**Published:** 2025-01-16

**Authors:** Zhenbin Bi, Wei Wang, Shanshen Gu, Yajing Zhou, Zhengchang Wu, Wenbin Bao, Haifei Wang

**Affiliations:** 1 Key Laboratory for Animal Genetics, Breeding, Reproduction and Molecular Design, College of Animal Science and Technology, Yangzhou University, Yangzhou, 225009 Jiangsu China; 2 Joint International Research Laboratory of Agriculture and Agri-Product Safety, The Ministry of Education of China, Yangzhou University, Yangzhou, 225009 Jiangsu China

**Keywords:** PED, virus-host protein interaction, virus infection, ubiquitination, antiviral response

## Abstract

Porcine epidemic diarrhoea virus (PEDV) is an enteric pathogen that causes acute diarrhoea, dehydration and high mortality rates in suckling pigs. Tripartite motif 8 (TRIM8) has been shown to play multiple roles in the host’s defence against viral infections. However, the functions of TRIM8 in regulating PEDV infection are still not well understood. In our study, we found a significant upregulation of TRIM8 following PEDV infection. We created TRIM8 knockout and overexpression cell lines and discovered that TRIM8 can inhibit PEDV replication within host cells. Co-immunoprecipitation assays revealed that TRIM8 directly interacts with the nucleocapsid protein (N) of PEDV, specifically within the coiled-coil structural domain of TRIM8. Furthermore, TRIM8 was shown to reduce the expression of the PEDV N protein in a dose-dependent manner. Mechanistically, TRIM8 inhibits the expression of PEDV N through K48-linked ubiquitin proteasome degradation. Transcriptomics analysis revealed that TRIM8 facilitates the expression of genes associated with several pathways, including the IL-17 signalling pathway, chemokine signalling pathway, and cytokine-cytokine receptor interaction. This suggests that TRIM8 plays a crucial role in boosting antiviral immune responses against PEDV infection. Our findings provide new insights into the functions and mechanisms of TRIM8 in regulating PEDV infection and highlight its potential as a molecular target for the prevention and control of this virus.

**Supplementary Information:**

The online version contains supplementary material available at 10.1186/s13567-024-01443-2.

## Introduction

Porcine epidemic diarrhoea (PED) is a highly contagious and acute enteric disease caused by the porcine epidemic diarrhoea virus (PEDV), which is an enveloped, single-stranded, positive-sense RNA virus belonging to the *Coronaviridae* family and the genus *Alphacoronavirus* [1].

The primary clinical signs of PED include acute watery diarrhoea, vomiting, dehydration, anorexia, and significantly high mortality rates. Pathological symptoms associated with PED include intestinal dilation filled with yellow foamy liquid, thinning of the bowel walls, severe atrophy of intestinal villi, and mesenteric congestion [2]. PED was first detected in the United Kingdom in 1971, and the virus was first isolated in Belgium in 1978. In 2010, variant strains of PEDV emerged in China, leading to significant economic losses for affected pig farmers [3].

The primary mode of transmission for PEDV is faecal-oral, often facilitated by contaminated feed, additives, and similar mediums. Additionally, research has indicated that PEDV can also be transmitted by air [4]. Furthermore, PEDV can occur both as a singular infection and as a coinfection with other viruses, including the porcine transmissible gastroenteritis virus and porcine delta coronavirus.

PEDV has had a severe impact, causing major economic losses within the global pig industry. As a result, it remains crucial to explore the interactions between PEDV and host cells to develop systematic prevention and control strategies for this virus.

The activation of antiviral immune responses and the degradation of virus proteins are crucial mechanisms that host cells use to control and combat virus infections. Recent studies have shown that various proteins, such as TARDBP and HNRNPA1, can activate interferon signalling to enhance the host’s antiviral defence, thereby restricting the replication of PEDV [5–7]. Additionally, a group of proteins has been identified that targets and degrades the nucleocapsid protein, envelop protein, and ORF3 protein of PEDV through direct interactions [8–10]. Ubiquitin-mediated degradation is one of the main mechanisms involved in the degradation of PEDV proteins during host-virus protein interactions.

The ubiquitin–proteasome system (UPS) is a crucial mechanism in eukaryotic cells that regulates protein degradation [11]. Ubiquitination modification is increasingly recognised as a significant strategy viral pathogens use to manipulate essential host factors for infection. The TRIM (tripartite motif-containing) family comprises a group of E3 ubiquitin ligases that play vital roles in the UPS and are involved in various cellular functions by regulating the levels of key proteins [12, 13].

For example, TRIM7 restricts different human enteric viruses by targeting viral membrane remodelling proteins for ubiquitination and proteasome-dependent degradation [14]. Similarly, TRIM22 can inhibit influenza A virus infection by promoting the ubiquitination and degradation of the viral nucleoprotein [15]. TRIM proteins play a crucial role in activating ubiquitin-dependent antiviral immune responses. For instance, TRIM31 interacts with MAVS to trigger an antiviral response through Lys 63-linked polyubiquitination [16]. Similarly, TRIM25 promotes Lys 63-linked ubiquitination of RIG-I, enhancing RIG-I’s ability to initiate antiviral signal transduction [17].

Additionally, TRIM8 is a significant regulator of TNF-α and IL-1β-triggered NF-κB activation, as well as the production of interferons triggered by viruses [18, 19]. However, the specific functions of TRIM8 in host cells responsive to PEDV infection remain largely unexplored.

To investigate the functions and underlying mechanisms of TRIM in response to PEDV infection, we knocked out and overexpressed TRIM8 in host cells to test its effects on viral infection. Our findings indicate that TRIM8 inhibits PEDV replication. Further mechanistic analysis revealed that TRIM8 directly interacts with the PEDV nucleoprotein and promotes its degradation through the ubiquitin–proteasome pathway. Additionally, transcriptomics analysis highlighted TRIM8’s involvement in immune responses to PEDV infection. These results provide new insights into the role and molecular mechanisms of TRIM8 in suppressing PEDV replication, offering valuable molecular targets and genetic resources for preventing and controlling PEDV infection.

## Materials and methods

### Cell lines, viruses, and antibodies

IPEC-J2 cells, alongside HEK293T cells and the PEDV CV777 strain, were preserved in our laboratory. Both IPEC-J2 cells and HEK293T cells were cultured in Dulbecco’s modified Eagle medium (Gibco, Thermo Fisher Scientific, Waltham, MA, USA) supplemented with 10% fetal bovine serum (Gibco, Thermo Fisher Scientific) and 1% penicillin/streptomycin (Invitrogen, Waltham, MA, USA). The cultures were maintained at 37 °C in an atmosphere with 5% CO_2_. The antibodies used in this study are detailed in Additional file 1.

### Plasmids and transfection

The full-length cDNA encoding TRIM8 and various truncated forms were cloned into the pCDNA3.1-Flag vector. The full-length cDNA encoding PEDV N, TRAF3 and TAK1 was also cloned into the pCDNA3.1 vector. Plasmids for HA-Ub and its mutants were constructed using standard molecular biology techniques. All transfections were carried out using jetPRIME (Polyplus, Illkirch, France) according to the manufacturer’s protocols. In brief, 20 μM siRNA or 2 μg vector (for 6-well plates only) was diluted in 200 μL of jetPRIME buffer and mixed by pipetting up and down. Next, 4 μL jetPRIME reagent was added to the mixture, which was then vortexed for 10 s, briefly spun down, and incubated for 15 min at room temperature. The transfection mix was then added to the cells in a culture medium, and the plate was gently rocked back and forth before being returned to the incubator.

### Generation of TRIM8 knockout IPEC-J2 cells

Three guide RNAs (Additional file 2) targeting the porcine TRIM8 gene were designed using ChopChop software [20]. The guide RNA sequences were ligated into the pGK1.2 vector. The recombinant vectors were transfected into IPEC-J2 cells using Lipofectamine 3000, following the manufacturer’s instructions. The transfected cells were selected with puromycin for seven days. The surviving cells were cultured and collected to assess the editing efficiency by PCR sequencing. Single clones of the knockout cells were isolated in 96-well plates using a limiting dilution assay. The expression of TRIM8 in the knockout cells was further analysed using western blotting.

### PEDV infection

Wild-type cells, TRIM8 knockout cells, and TRIM8 overexpression cells were seeded in a 6-well plate and infected with PEDV at MOI = 1. After 2 h of adsorption, the infected cells were washed three times with PBS and then cultured in a fresh medium. After 24 h of incubation, the cells were collected and subjected to three cycles of freezing and thawing to isolate the PEDV genome using Trizol reagent (Thermo Scientific, Waltham, MA, USA), following the manufacturer’s guidelines. The quantities of PEDV in the cells were determined by measuring the expression of the PEDV M gene using qRT-PCR.

### Quantitative real-time PCR (qRT-PCR)

Total RNA was extracted and assayed from cells infected with PEDV at 12, 24, and 48 h, as well as from uninfected cells at 0 h, using the Trizol method. CDNA was obtained through reverse transcription with the HiScript III RT SuperMix for qPCR kit (Vazyme, Nanjing, China). qRT-PCR was conducted using the AceQ Universal SYBR qPCR Master Mix kit (Vazyme), with GAPDH as the reference gene. The relative quantification of gene expression levels was calculated using the 2^−ΔΔCt^ method. The PCR products specific to PEDV were sequenced by Sangon Biotech (Shanghai, China) Co. Ltd., and sequence alignment was performed using BLAST. The primers are listed in Additional file 3.

### Homology modeling

A homology model of TRIM8 (F1S855_PIG) and PEDV N (NCAP_PEDV7) was created using SWISS-MODEL online software [21]. The completes sequence of TRIM8 (F1S855) and PEDV N(Q07499) used for homology modelling were obtained from Uniprot [22]. To analyse the interfaces between the two proteins, we used [23]. PEDV N comprises two identical amino acid sequences, which should be examined individually in relation to TRIM8 for docking site analysis due to the positioning in different docking domains. In this context, the TRIM8 chain is referred to as chain C, while the two chains of the PEDV N protein are labelled as chain A and chain B. The docking results were visualised using PyMOL 2.3.2.

### Western blot

Cells were lysed using a cell lysate, and the protein concentration was quantified using the BCA kit (Beyotime Biotechnology, Shanghai, China). The proteins were denatured and loaded onto a 10% SDS-PAGE for electrophoresis. After electrophoresis, the proteins were transferred to 0.22-μm polyvinylidene difluoride membranes (Millipore, MA, USA). The membranes were blocked with 5% skim milk powder for 2 h and eluted, followed by overnight incubation at 4 °C with primary antibodies. Afterwards, the membranes were incubated with the corresponding secondary antibodies, and the protein bands were detected using an enhanced chemiluminescence detection system (Bio-Rad, Hercules, CA, USA).

### Indirect immunofluorescence assays

Cells were fixed using 4% paraformaldehyde and treated with 0.05% Triton X-100. After that they were blocked with 5% bovine serum albumin for 2 h. The cells were then incubated overnight at 4 °C with primary antibodies. Following this, the cells were rinsed thrice with PBS and incubated with fluorescently labelled secondary antibodies for 1 h in the dark. Finally, fluorescent images were captured using a laser-scanning confocal immunofluorescence microscope (Carl Zeiss AG, Oberkochen, Germany).

### Co-immunoprecipitation (Co-IP) assay

Transfection was conducted on 293 T cells, and cell samples were collected 48 h later for a Co-IP assay, according to the protein A/G magnetic bead protocol (MCE, NJ, USA). The complexes were incubated with the lysate supernatant at 4 °C overnight. Afterwards, the magnetic beads were washed with the washing buffer four times, and SDS-PAGE sampling buffer was added. The eluted proteins were then analysed using SDS-PAGE, and the corresponding antibodies were applied for western blot analysis.

### RNA-seq and data analysis

Cells collected from the assay were subjected to RNA-seq. Total RNA was isolated from the cell samples using TRIzol reagent, following the manufacturer’s instructions. The sequencing library was prepared according to the methods outlined in our previous study [24]. Raw reads were filtered with SOAPnuke (v1.4.0) software to eliminate low-quality reads. The cleaned reads were then aligned to the genome assembly Sscrofa11.1 using Bowtie2 (v2.2.5) [25]. Gene expression levels for each sample were calculated using RESM (v1.2.8) [26]. Differentially expressed genes between the different groups (PEDV-infected group versus uninfected control group, PEDV-infected TRIM8 overexpression group versus PEDV-infected group) were identified using DESeq [27]. Genes with an adjusted *P*-value ≤ 0.05 and |log_2_ fold change|≥ 1 were classified as differentially expressed. The RNA-seq data in this study has been submitted to the NCBI Sequence Read Archive under the accession number PRJNA1180182.

### Functional annotation and enrichment analysis

Gene ontology annotation and KEGG pathway enrichment analyses were conducted on the differentially expressed genes using the clusterProfiler software package within the R programming environment [28]. Biological terms and pathways with an adjusted *P*-value ≤ 0.05 were regarded as significantly enriched.

### Statistical analysis

Excel software was used to compare the differences between the experimental and control groups. Results are presented as the mean ± standard deviation (SD) from three replicates in each group. A two-sided Student’s *t* test was used to analyse the differences between the two groups. Statistical significance is indicated as follows: ^*^ *P* < 0.05, ^**^ *P* < 0.01.

## Results

### TRIM8 is upregulated by PEDV infection

**Figure 1:**
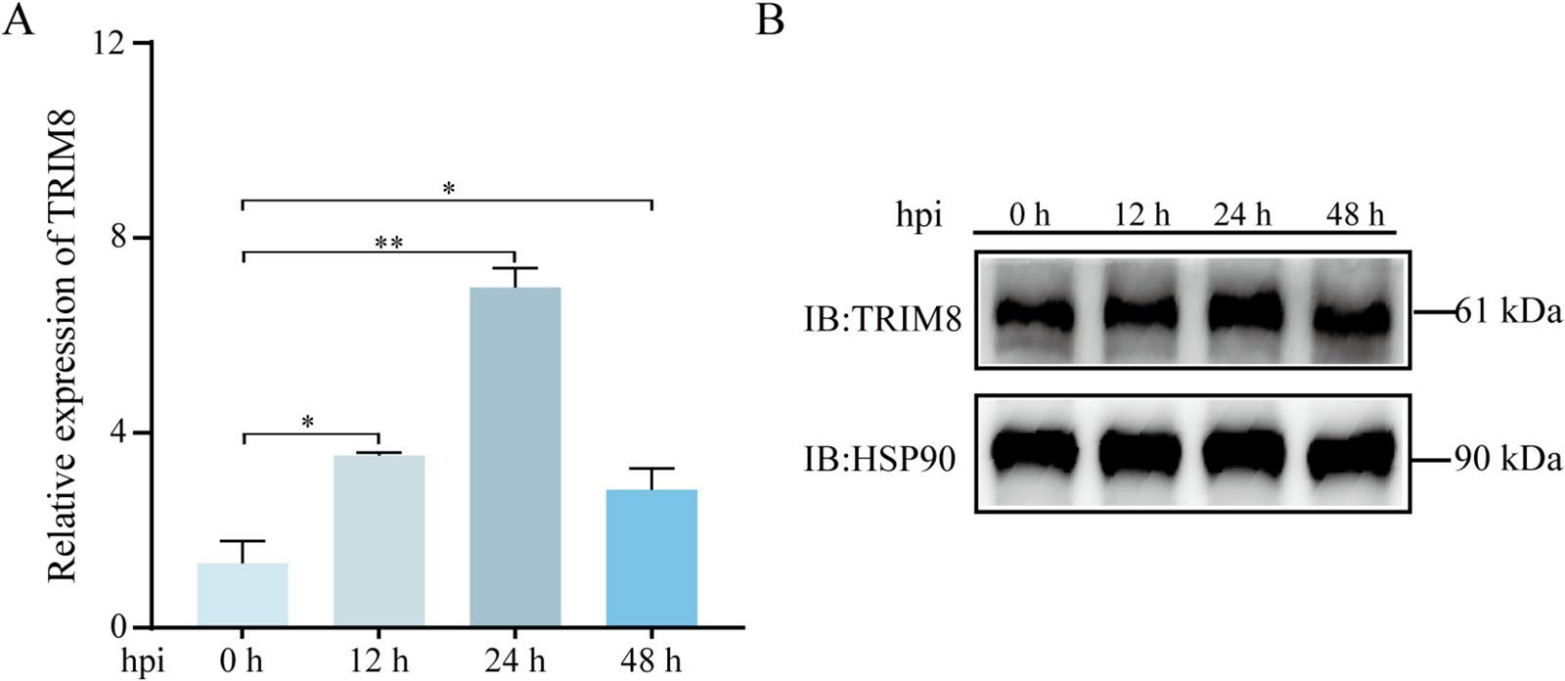
**PEDV infection induces the expression of TRIM8 in IPEC-J2 cells.** Relative mRNA **A** and protein **B** expression levels of TRIM8 at different time points (0, 12, 24, 48 h). hpi: hours post-infection. *P < 0.05, ** *P* < 0.01.

To explore and investigate the relationship between TRIM8 and PEDV infection, we measured the expression of TRIM8 in PEDV-infected IPEC-J2 cells using qRT-PCR and western blot analysis. The results showed that the expression level of the TRIM8 gene was significantly upregulated at 12, 24, and 48 h of infection, with the highest level observed at 24 h post-infection (Figure 1A). Additionally, the western blot assay confirmed the changes in TRIM8 expression at different infection time points following infection (Figure 1B). These findings indicate that PEDV infection can stimulate the expression of TRIM8 in host cells.

### TRIM8 suppresses PEDV replication in host cells

**Figure 2:**
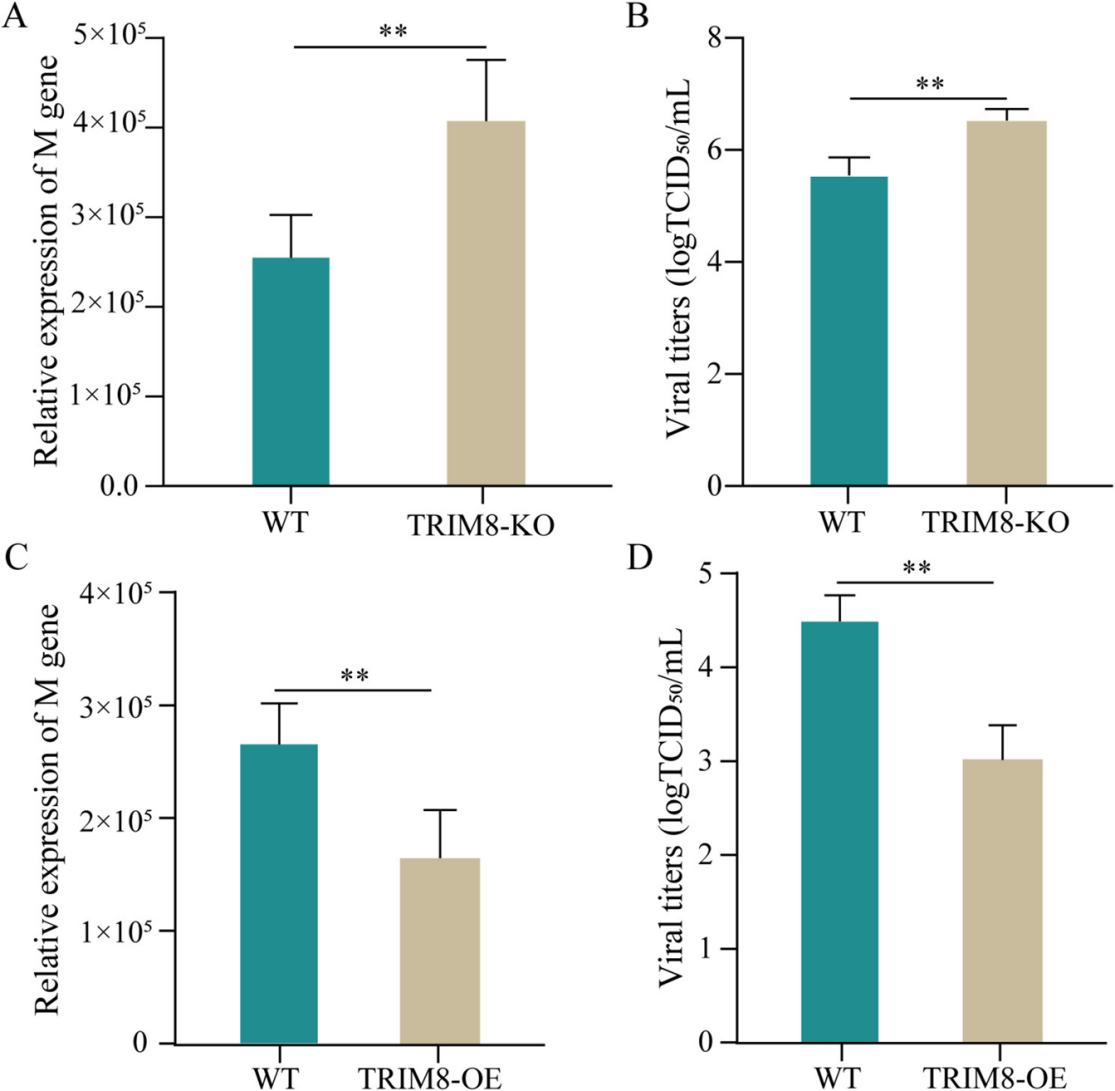
**TRIM8 inhibits PEDV infection in host cells. **Relative expression of PEDV M gene **A** and virus titres **B** in PEDV-infected TRIM8 knockout and wild-type cells. Relative expression of PEDV M gene **C** and virus titres **D** in PEDV-infected TRIM8 overexpression and wild-type cells. WT: wild-type cells; TRIM8-KO: TRIM8 knockout cells; TRIM8-OE: TRIM8 overexpression cells. *P < 0.05, **P < 0.01.

To investigate the role of TRIM8 in the cellular response to PEDV infection, we established TRIM8 knockout cells (Additional file 4) and infected them with PEDV. The results indicated that the genome copies of PEDV and the virus titres in TRIM8 knockout cells were significantly lower than those in wild-type cells (Figures 2A and B). To further validate the function of TRIM8, we constructed TRIM8 overexpression cells (Additional file 5) and found that the PEDV genome copy numbers were significantly lower in the TRIM8 overexpression group compared to the control group (Figure 2C). Additionally, the TCID_50_ assay demonstrated that the virus titres were markedly reduced in the TRIM8 overexpression group (Figure 2D). These results suggest that TRIM8 plays a crucial role in inhibiting PEDV infection in host cells.

### TRIM8 directly interacts with PEDV N protein

**Figure 3:**
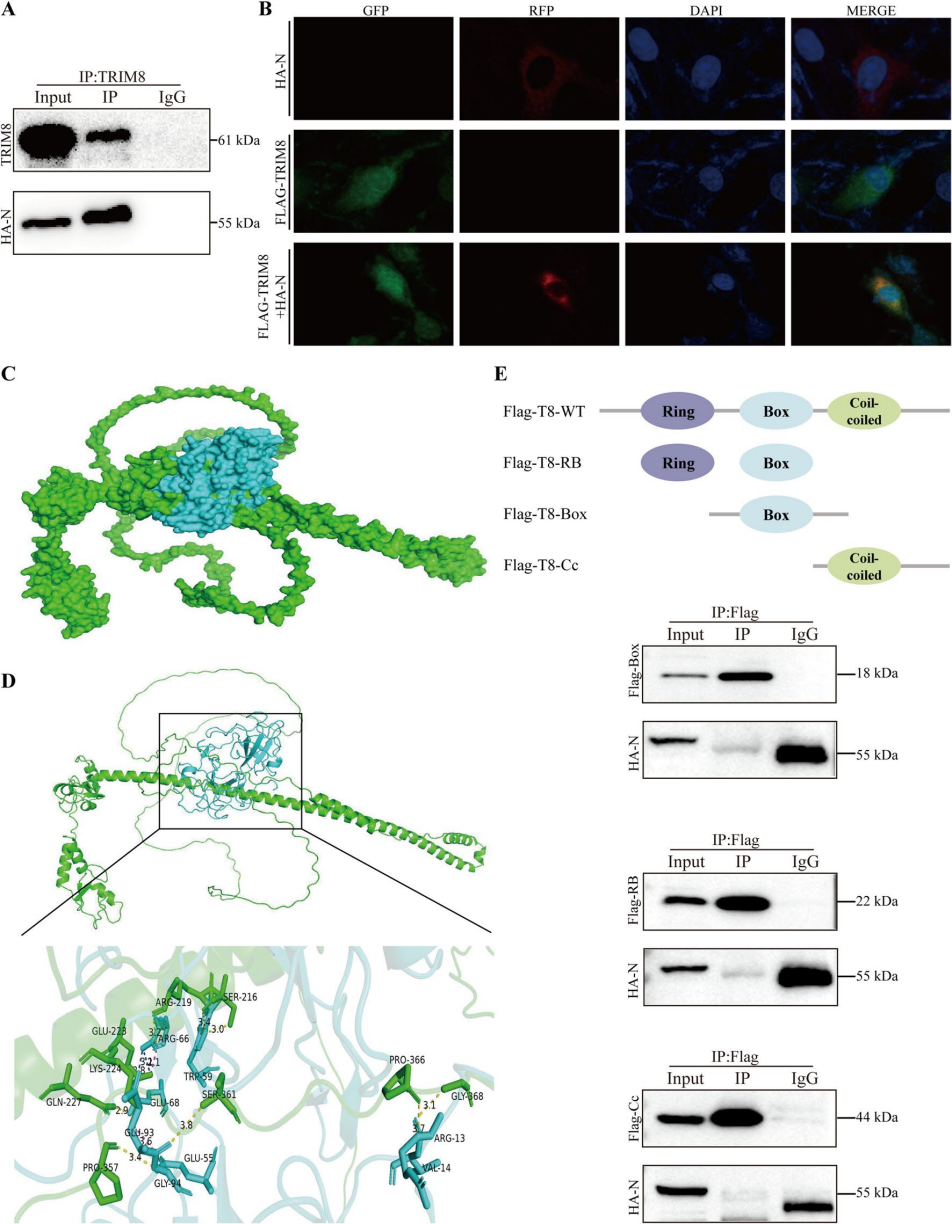
**TRIM8 colocalises and interacts with PEDV N protein.** **A** Co-IP of HEK293T cells expressing TRIM8 and HA-tagged PEDV N proteins. **B** Fluorescence co-localization of FLAG-tagged TRIM8 and HA-tagged N proteins in HEK293T cells. Staining was performed using DAPI, GFP, and RFP, respectively. Scale bars, 10 μm. **C** Prediction of the interaction domain between the PEDV N protein and TRIM8 by PyMOL software. Green and cyan indicates TRIM8 and PEDV N protein, respectively. **D** Key binding sites predicted for the interaction domain of PEDV N and TRIM8 proteins. Ribbon representations are coloured according to the proteins: TRIM8 (green) and PEDV N (cyan). **E** Co-IP of FLAG-tagged TRIM8 and its truncated mutants with HA-tagged PEDV N proteins in HEK293T cells. WT: full length of TRIM8; RB: RING and B box domains of TRIM8; Box: B box domain of TRIM8; CC: coiled coil domain of TRIM8.

Host cellular proteins can target and degrade viral proteins to inhibit virus replication, and among these, the PEDV N protein is one of the most targeted [29, 30]. To better understand how TRIM8 suppresses PEDV replication, we conducted a Co-IP to determine if TRIM8 directly interacts with the PEDV N protein. The results indicated that TRIM8 does indeed interact with HA-tagged PEDV N protein (Figure 3A). Following this, we examined the colocalisation of TRIM8 and N using confocal microscopy in cells expressing TRIM8 fused with green fluorescent protein and PEDV N fused with mCherry protein. The observations revealed that TRIM8 and N proteins were colocalised in the cytoplasm (Figure 3B).

The docking site analysis initially found no definitive evidence of a docking site between chains C and chain B. However, interactions between these two chains later became a key focus of our study. In contrast, no apparent docking site was observed between chains A and B. As a result, our analysis primarily concentrated on detailing the docking sites between chains C and B, with TRIM8 chain C depicted in green and the PEDV N terminal of chain A/chain B represented in cyan (Figure 3C). Based on multiple-sequence alignments, the corresponding residues in TRIM8 are K224, Q227, S361, G368, S216, R219, E223, P357, and P366. As homology modelling indicates, these residues may form a pocket associated with the TRIM8 PEDV-N terminus (Figure 3D).

Based on the predicted interaction sites, the PEDV N protein likely interacted with TRIM8, specifically within the coiled-coil (cc) structural domain of TRIM8. To investigate this further, we constructed a series of Flag-tagged TRIM8 expression vectors, each encoding distinct truncated structural domains. We then performed co-immunoprecipitation (Co-IP) by co-transfecting vectors expressing Flag-tagged TRIM8 and HA-tagged PEDV N. The results conclusively demonstrated an interaction between the TRIM8-cc structural domain and the PEDV N protein, confirming a physical association between the two domains (Figure 3E).

### TRIM8 degrades PEDV N protein via the ubiquitin–proteasome system

**Figure 4:**
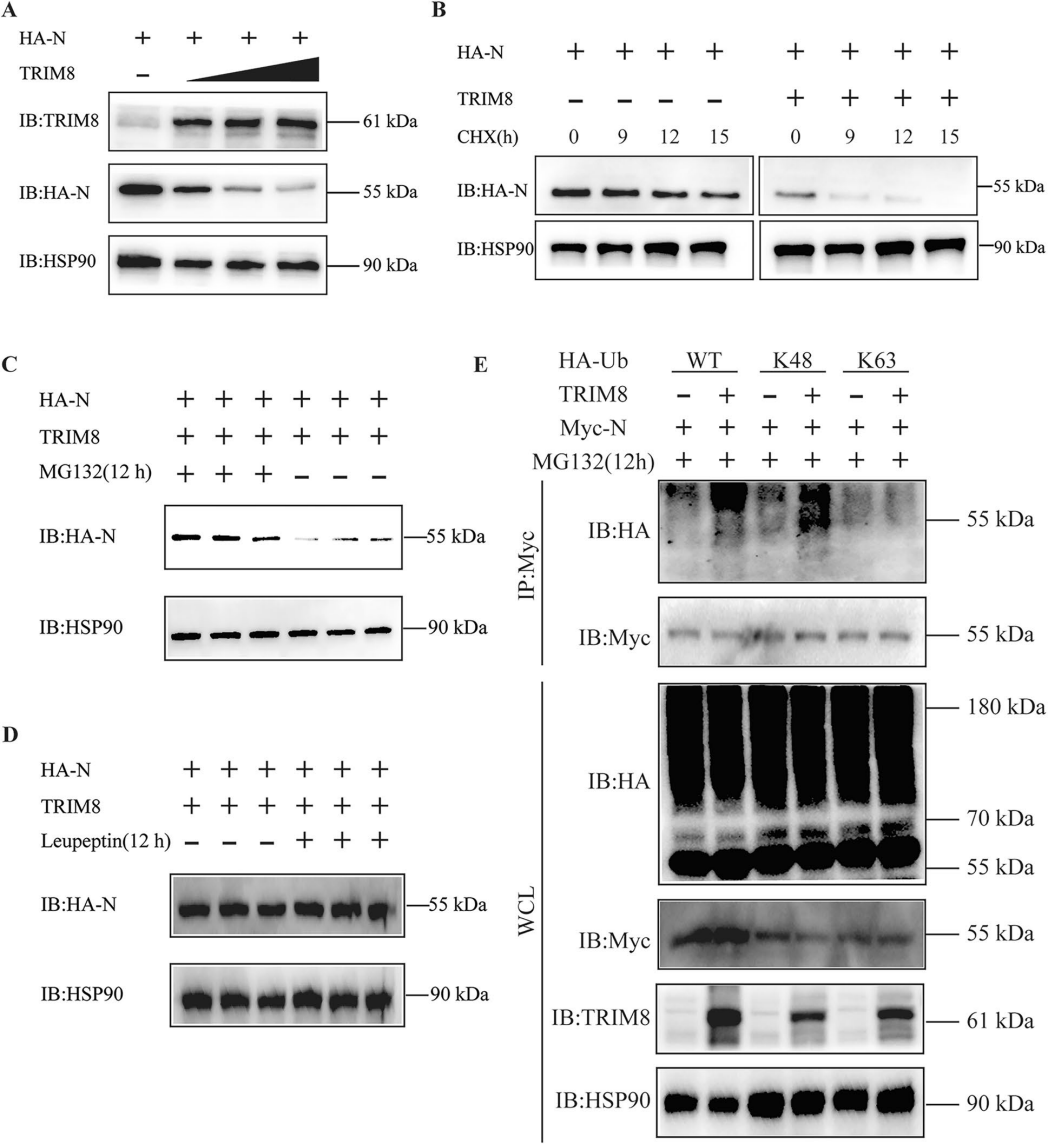
**TRIM8 catalyses PEDV N protein degradation via K48-linked ubiquitination.** **A** Co-IP and IB analysis of HEK293T cells expressing HA-tagged PEDV N protein and increasing amounts of TRIM8. **B** IB analysis of cells expressing HA-tagged PEDVN with or without TRIM8 under CHX treatment for different time points (0, 9, 12, 15 h). CHX: Cycloheximide. **C** IB analysis of cells expressing HA-tagged PEDV N under the treatment of proteasome inhibitor MG132 for 12 h. **D** IB analysis of cells expressing HA-tagged PEDV N under the treatment of autophagy-lysosome inhibitor Leupeptin for 12 h. **E** Co-IP and IB analysis of MG132-treated cells expressing MYC-tagged PEDV N, with or without TRIM8, HA-tagged wild-type ubiquitin, mutant ubiquitin K48 and K63. WCL: whole cell lysates.

To investigate the effects of TRIM8 on the expression of the PEDV N protein, we conducted co-transfection experiments where TRIM8 was introduced alongside the PEDV N protein. Our results showed that TRIM8 significantly reduced the expression of the PEDV N protein in a dose-dependent manner (Figure 4A). To further understand the timing of this inhibition, we conducted an experiment to inhibit protein synthesis in the cells. We observed that the expression of the PEDV N protein was not significantly affected in cells lacking TRIM8 expression (Figure 4B). In contrast, cells overexpressing TRIM8 exhibited a notable decrease in the expression of the PEDV N protein (Figure 4B). The data indicated that TRIM8 did not affect the synthesis of the PEDV N protein but may inhibit its expression by promoting protein degradation.

The ubiquitin–proteasome system and the autophagy-lysosomal pathway are key cellular mechanisms for mediating protein degradation in eukaryotic cells [11]. Inhibition assays of these two pathways showed that inhibiting the proteasome significantly reduced the degradation of the PEDV N protein induced by TRIM8 overexpression (Figure 4C). In contrast, inhibiting the autophagy-lysosomal pathway did not result in significant changes in the expression of the PEDV N protein (Figure 4D). This suggests that TRIM8 mediates the degradation of the PEDV N protein primarily through the proteasome system rather than the lysosomal pathway.

TRIM8, an E3 ubiquitin ligase, is capable of mediating protein degradation through multiple lysine residues (K48, K63, K11, and K27)-linked ubiquitination [31]. We conducted a Co-IP assay to explore the type of TRIM8-mediated polyubiquitination of the PEDV N protein. Our observations revealed that TRIM8 facilitates the polyubiquitination of the PEDV N protein in the presence of wild-type ubiquitin (HA-WT) and mutant ubiquitin (HA-K48) but not in the presence of the mutant ubiquitin (HA-K63) (Figure 4E). These findings indicate that TRIM8 regulates the expression of PEDV N through the K48-linked ubiquitin–proteasome degradation.

### Transcriptomics analysis of TRIM8 overexpressing cells infected with PEDV

To investigate the impact of TRIM8 overexpression on the expression patterns of genes associated with PEDV infection, we conducted a transcriptomics analysis on PEDV-infected cells with TRIM8 overexpression and wild-type cells. We obtained a total of 206.93 million clean reads across all samples, with 22.99 million clean reads per sample (Additional file 6).

**Figure 5:**
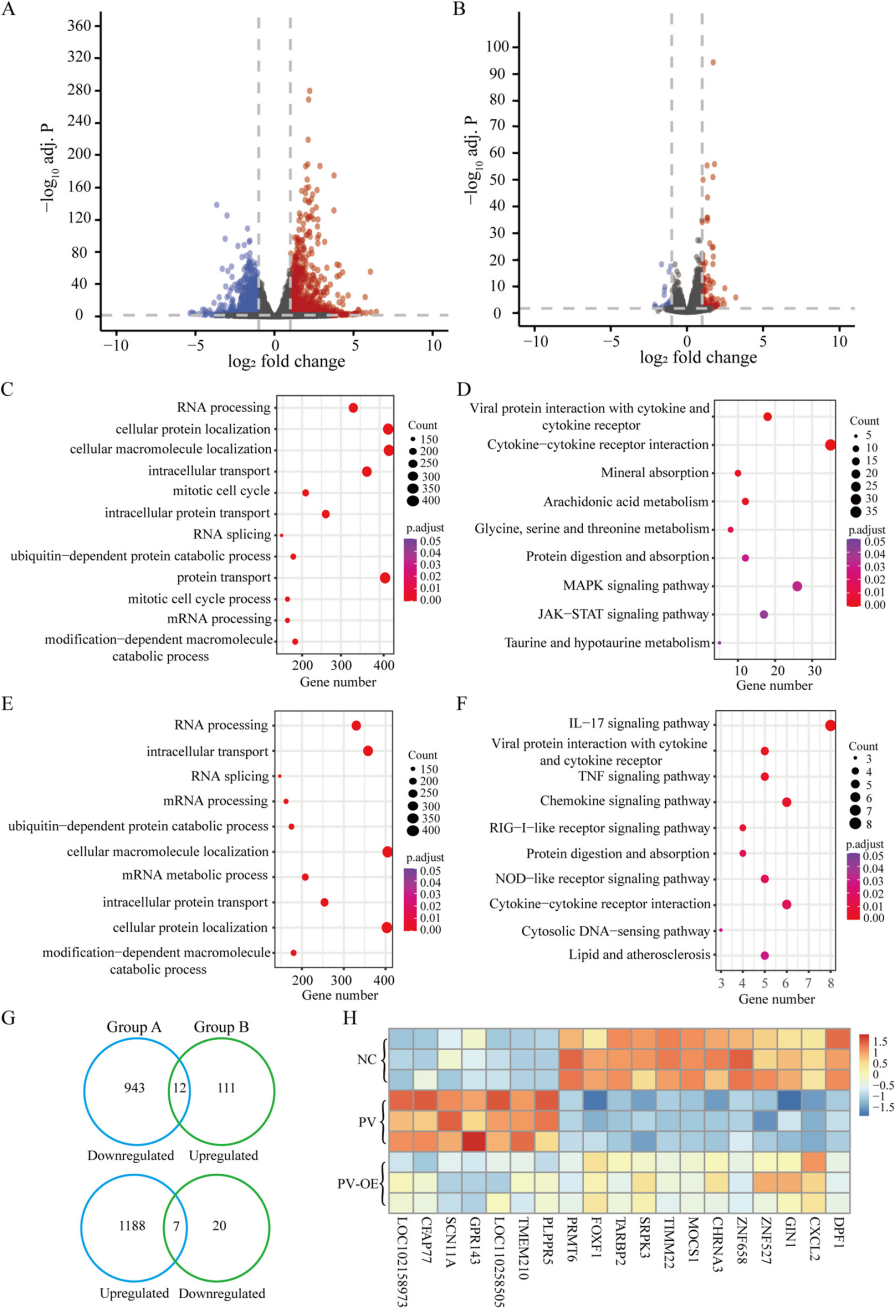
**Changes in the transcriptomes of PEDV-infected TRIM8 overexpression and control cells. A** Volcano plot of differentially expressed genes between PEDV-infected and uninfected cells. **B** Volcano plot of differentially expressed genes between PEDV-infected TRIM8 overexpression and wild-type cells. **C-D** GO annotation and KEGG enrichment analysis for differentially expressed genes are shown in **A**. **E–F** GO annotation and KEGG enrichment analysis for differentially expressed genes shown in **B**. **G** Intersected genes between different groups. Group A represents differentially expressed genes shown in **A**; Group B represents differentially expressed genes shown in **B**. **H** Expression profiles of the intersected genes shown in **G**.

Hierarchical clustering and principal component analyses revealed a clear separation between the samples from different groups (Additional file 7). Differential expression analysis revealed a total of 2,153 differentially expressed genes between PEDV-infected and uninfected cells. Among these, 1,195 genes were upregulated, while 958 were downregulated (Figure 5A; Additional file 8).

Additionally, the analysis identified 153 differentially expressed genes between PEDV-infected TRIM8 overexpression cells and wild-type cells, with 126 genes upregulated and 27 genes downregulated (Figure 5B; Additional file 9). We randomly selected four upregulated and four downregulated genes to validate their expression changes. Our analysis revealed that the expression patterns of these genes were highly consistent between the RNA-seq and qRT-PCR analyses (Additional file 10). This consistency indicates high reliability and accuracy in our differential gene expression analysis.

Functional enrichment analysis of differentially expressed genes between PEDV-infected and uninfected cells revealed significant enrichment in functional terms, including RNA processing and cellular protein localisation (Figure 5C; Additional file 11), as well as in the pathways, including cytokine-cytokine receptor interaction and MAPK signalling (Figure 5D; Additional file 12). A functional enrichment analysis of differentially expressed genes between PEDV-infected TRIM8 overexpression and control cells showed the following functional terms were enriched: RNA processing and intracellular transport (Figure 5E; Additional file 13), and in the pathways including the IL-17 signalling pathway, the chemokine signalling pathway, and the cytokine-cytokine receptor interaction (Figure 5F; Additional file 14).

The intersection analysis of differentially expressed genes across different groups identified 12 genes, including CXCL2 and ZNF527, downregulated in the PEDV-infected group and upregulated in the PEDV-infected TRIM8 overexpression group. Additionally, 7 genes, such as PLPPR5 and TMEM210, were found to be upregulated in the PEDV-infected group and downregulated in the PEDV-infected TRIM8 overexpression group (Figure 5G). Functional enrichment analysis showed that these genes were significantly enriched in ribonucleoprotein complex biogenesis. This category included three genes (TARBP2, ZNF658, and SRPK3) that were upregulated in PEDV-infected TRIM8 overexpression cells when compared to the PEDV-infected cells (Figure 5H; Additional file 15).

## Discussion

In recent years, TRIM proteins have been recognised for their important roles in protecting the host against viral infections. They achieve this by directly counteracting key proteins involved in the viral cycle or regulating signal transduction pathways initiated by innate immune sensors. Additionally, TRIM proteins are linked to virus-induced autophagy and the clearance of viruses through autophagy mechanisms [32, 33]This study specifically examined the roles and mechanisms of TRIM8 during PEDV infection. Our findings indicated that TRIM8 plays an inhibitory role in the replication of PEDV within host cells by degrading the virus's N protein.

TRIM proteins can directly target viral proteins to exert antiviral effects, either by inhibiting their functions or degrading the interacting proteins. For instance, TRIM52 targets the NS2A protein of the Japanese encephalitis virus and degrades it, thereby inhibiting virus replication [34]. Similarly, TRIM22 ubiquitinates the NS5A in a concentration-dependent manner, which also hinders hepatitis C virus replication [35]. Furthermore, TRIM41 ubiquitinates the nucleoprotein of the vesicular stomatitis virus, leading to its degradation and subsequently inhibiting virus replication [36]. In our study, we revealed for the first time that TRIM8 directly interacts with the PEDV N protein and degrades it to inhibit PEDV replication. We also demonstrated that TRIM8 degrades the N protein of PEDV via the ubiquitin–proteasome system. Our findings highlight new roles for TRIM8 in regulating PEDV infection, expanding our understanding of the antiviral activities of TRIM proteins.

TRIM proteins play versatile roles in the antiviral immune responses of host cells during viral infections. They can activate the functions of interferon regulatory factors and NF-κB through a series of phosphorylation and ubiquitination processes, which subsequently induce the production of type I interferons and proinflammatory cytokines [37]. TRIM8, in particular, regulates TNFα and IL-1β-triggered NF-κB activation by targeting TAK1 for K63-linked polyubiquitination [18]. Additionally, TRIM 8 protects phosphorylated IRF7, promoting the production of type I interferons [19]. We observed an upregulation of TRIM8 following PEDV infection and identified differentially expressed genes associated with TRIM8 expression in response to this infection. Furthermore, these differentially expressed genes were primarily enriched in pathways, including the IL-17 signalling pathway, viral protein interaction with cytokine and cytokine receptors, and cytokine-cytokine receptor interaction. Most of the genes involved were upregulated. These findings highlight the role of TRIM8 in activating the antiviral immune response to combat PEDV infection.

The CXCL2 gene and members of the zinc finger protein (ZNF) family, specifically ZNF527 and ZNF658, were found to be upregulated in TRIM8 overexpression cells infected with PEDV compared to wild-type cells infected with the same virus. During viral infection, the expression of chemokines in epithelial cells plays a crucial role in the tissue inflammatory response mediated by various cell types [38]. The zinc finger protein family is involved in host-virus interactions, where ZNF proteins inhibit viral replication in host cells by recognising viral genomes. This family of proteins is characterised by their diverse structures and coordination with zinc ions, with TRIM proteins also classified as zinc finger proteins. Specifically, zinc finger antiviral proteins contain four tandem CCCH-type zinc finger motifs at their N-terminus and have been shown to act as antiviral agents by recognising viral RNA [39]. Research has indicated that the activity of zinc finger antiviral proteins can be antagonised by the PEDV N protein, which interacts with these antiviral proteins [40]. These findings indicated that TRIM8 may facilitate the activity of zinc finger antiviral proteins by degrading the PEDV N protein.

TARBP2 is an RNA binding protein that is crucial in regulating mRNA stability and processing microRNAs [41, 42]. Research has shown that TARBP2 is often dysregulated in various cancers, including prostate cancer and colorectal cancer [43]. Additionally, TARBP2 has multiple functions in modulating virus infections and the host immune response. It has been identified as a cellular protein that aids in replicating the human immunodeficiency virus by inhibiting protein kinase activation [44]. Furthermore, TARBP2 regulates the antiviral response by targeting the mitochondrial antiviral signalling protein [45]. In our study, we observed differential expression of TARBP2 following PEDV infection, suggesting its potential role in the cellular response to this virus. The mechanisms by which TARBP2 regulates PEDV infection and its specific functions merit further investigation.

Our study has several limitations. First, the use of the CRISPR/Cas9 system to create TRIM8 knockout cells may lead to off-target effects, which can result in unintended genomic mutations and disrupt gene function. Second, while we have demonstrated the antiviral roles of TRIM8 in vitro, these conditions do not fully replicate the complex microenvironment and various cell types in the intestine. To provide more robust evidence for the role of TRIM8 in inhibiting PEDV replication, it will be important to use gene-edited pigs as an in vivo model to further validate our findings.

In summary, our findings demonstrate that TRIM8 directly interacts with the PEDV N protein and regulates its expression of PEDV N through K48-linked ubiquitin–proteasome degradation. Additionally, TRIM8 enhances gene expression in the antiviral immune response, thereby inhibiting PEDV infection. This study provides new insights into the functions and mechanisms of TRIM8 in response to PEDV infection, highlighting its potential as a molecular target for combatting PEDV.

## Supplementary Information

**Additional file 1. Antibodies used in this study.**

**Additional file 2. SgRNA sequence targeting porcine TRIM8 gene.**

**Additional file 3. Primes for qRT-PCR.**

**Additional file 4. Generation of TRIM8 knockout IPEC-J2 cells by CRISPR/Cas9 technology.**

**Additional file 5. TRIM8 expression at mRNA (left) and protein (right) levels in TRIM8 overexpression and control cells.**

**Additional file 6. Statistics for RNA-seq data of each sample.**

**Additional file 7. Hierarchical clustering (A) and principal component (B) analyses of the samples used in RNA-seq.**

**Additional file 8. Differentially expressed genes between PEDV-infected and uninfected control groups.**

**Additional file 9. Differentially expressed genes between PEDV-infected TRIM8 overexpression and wild-type groups.**

**Additional file 10. Validation of differentially expressed genes from RNA-seq data by qRT-PCR.**

**Additional file 11. GO annotation for differentially expressed genes between PEDV-infected and uninfected control groups.**

**Additional file 12. KEGG enrichment for differentially expressed genes between PEDV-infected and uninfected control groups.**

**Additional file 13. GO annotation for differentially expressed genes between PEDV-infected TRIM8 overexpression and wild-type groups.**

**Additional file 14. KEGG enrichment for differentially expressed genes between PEDV-infected TRIM8 overexpression and wild-type groups.**

**Additional file 15. GO annotation for the intersected differentially expressed genes between different groups.**

## Data Availability

All of the data analysed in this work are included in this published article. The raw data generated in this study are available upon reasonable request.
