# Supplementary material for: TRIM8 inhibits porcine epidemic diarrhoea virus replication by targeting and ubiquitinately degrading the nucleocapsid protein
Source: Vet Res. 2025 Jan 16;56:14. doi: 10.1186/s13567-024-01443-2 (PMC11740423; doi:10.1186/s13567-024-01443-2)
Supplement: Supplementary file 1 — Additional file 1. Antibodies used in this study. [file 13567_2024_1443_MOESM1_ESM.docx]

**Additional file 1. Antibodies used in this study.**

| Antibodies and fluorescent dyes | Source | Application | Cat No. |
| --- | --- | --- | --- |
| PEDV-N | Medgene Labs | Western blot, IFA | DA0124 |
| TRIM8 | Proteintech | Western blot, Co-IP | 27463-1-AP |
| Flag-tag | Proteintech | Western blot, Co-IP, IFA | 80010-1-RR |
| Ha-tag | Proteintech | Western blot, Co-IP, IFA | 51064-2-AP |
| Myc-tag | Abbkine | Western blot, Co-IP | ABT2061 |
| HSP90 | Proteintech | Western blot | 60318-1-Ig |
| GAPDH | Proteintech | Western blot | 60004-1-Ig |
| IgG | Proteintech | Co-IP | 30000-0-AP |
| FITC, Goat Anti-Mouse IgG | Abbkine | IFA | A22110 |
| FITC, Goat Anti-Rabbit IgG | Abbkine | IFA | A22120 |
| CoraLite594 – conjugated Goat Anti-Mouse IgG(H+L) | Proteintech | IFA | SA00013-3 |
| CoraLite594 – conjugated Goat Anti-Rabbit IgG(H+L) | Proteintech | IFA | SA00013-4 |
