# Supplementary material for: TRIM8 inhibits porcine epidemic diarrhoea virus replication by targeting and ubiquitinately degrading the nucleocapsid protein
Source: Vet Res. 2025 Jan 16;56:14. doi: 10.1186/s13567-024-01443-2 (PMC11740423; doi:10.1186/s13567-024-01443-2)
Supplement: Supplementary file 2 — Additional file 2. SgRNA sequence targeting porcine TRIM8 gene. [file 13567_2024_1443_MOESM2_ESM.docx]

**Additional file 2. SgRNA sequence targeting porcine TRIM8 gene.**

| sgRNA ID | Sequence (5'-3') |
| --- | --- |
| sgRNA1 | CCTGAAGCTCACTAACATCG |
| sgRNA2 | CTACCGCCTTTACCACTGCG |
| sgRNA3 | GGGACATTCGGTGTGCGACG |
