# Supplementary material for: TRIM8 inhibits porcine epidemic diarrhoea virus replication by targeting and ubiquitinately degrading the nucleocapsid protein
Source: Vet Res. 2025 Jan 16;56:14. doi: 10.1186/s13567-024-01443-2 (PMC11740423; doi:10.1186/s13567-024-01443-2)
Supplement: Supplementary file 3 — Additional file 3. Primes for qRT-PCR. [file 13567_2024_1443_MOESM3_ESM.docx]

**Additional file 3. Primes for qRT-PCR.**

| Gene name | Primer Sequence (5’→3’) | Fragment Size (bp) |
| --- | --- | --- |
| PEDV M | F: AGGTCTGCATTCCAGTGCTT | 216 |
|  | R: GGACATAGAAAGCCCAACCA |  |
| TRIM8 | F: AATCCTAATGGACAGGACCCAG | 192 |
|  | R: CTCACGCCACAAGGGTACAG |  |
| HSPA6 | F: TCCTAGTGGGTGGCTCTACC | 159 |
|  | R: CCTTCTCACACTTGTCCCCC |  |
| ISG15 | F: CGGCAATGTGCTTCAGGATG | 105 |
|  | R: AGGATGCTCAGTGGGTCTCT |  |
| IFI6 | F: GGTATCGCTCTTCTTGTGCT | 72 |
|  | R: GAGCGTCTTCTTTTGTCTGTCT |  |
| MX1 | F: GTGGAGAAAAGTCACAAAACAGGGC | 288 |
|  | R: TTTGCCCTTCCATTCGTCTTCT |  |
| MTD1 | F: ATGGACCCCAACTGCTCCTGC | 186 |
|  | R: TCAGGCACAGCAGCTACACTT |  |
| LOC100526184 | F: CCCATCAGAGCCAGAGGTCG | 70 |
|  | R: CTTGGCTCTCACAGACTGGAA |  |
| ALDOB | F: TGCTGGGAATCAAGTTAGACCA | 171 |
|  | R: GGGATGGACACTGCTTGTCA |  |
| TMEM210 | F: GAGTTCCGTCCAGAGGTGTG | 279 |
|  | R: CCAGGGCATGTTTCACCCTT |  |
| GAPDH | F: ACATCATCCCTGCTTCTACTGG | 188 |
|  | R: CTCGGACGCCTGCTTCAC |  |
