# Supplementary material for: TRIM8 inhibits porcine epidemic diarrhoea virus replication by targeting and ubiquitinately degrading the nucleocapsid protein
Source: Vet Res. 2025 Jan 16;56:14. doi: 10.1186/s13567-024-01443-2 (PMC11740423; doi:10.1186/s13567-024-01443-2)
Supplement: Supplementary file 4 — Additional file 4. Generation of TRIM8 knockout IPEC-J2 cells by CRISPR/Cas9 technology. [file 13567_2024_1443_MOESM4_ESM.docx]

**
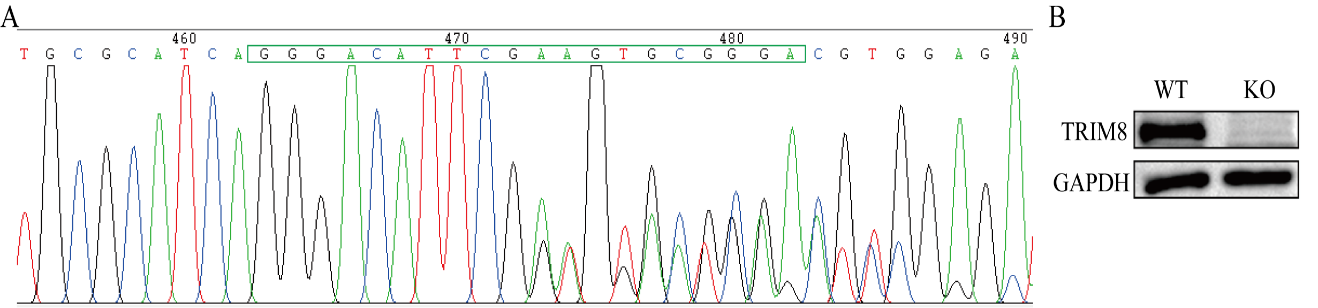
**

**Additional file 4. Generation of TRIM8 knockout IPEC-J2 cells by CRISPR/Cas9 technology.** (A) PCR sequencing of TRIM8 knockout cells. The green rectangle indicates the target sites by sgRNA3. (B) Western blotting of TRIM8 expression in TRIM8 knockout and wild type cells. KO: TRIM8 knockout cells; WT: wild type cells.
