# Supplementary material for: TRIM8 inhibits porcine epidemic diarrhoea virus replication by targeting and ubiquitinately degrading the nucleocapsid protein
Source: Vet Res. 2025 Jan 16;56:14. doi: 10.1186/s13567-024-01443-2 (PMC11740423; doi:10.1186/s13567-024-01443-2)
Supplement: Supplementary file 5 — Additional file 5. TRIM8 expression at mRNA (left) and protein (right) levels in TRIM8 overexpression and control cells. [file 13567_2024_1443_MOESM5_ESM.docx]

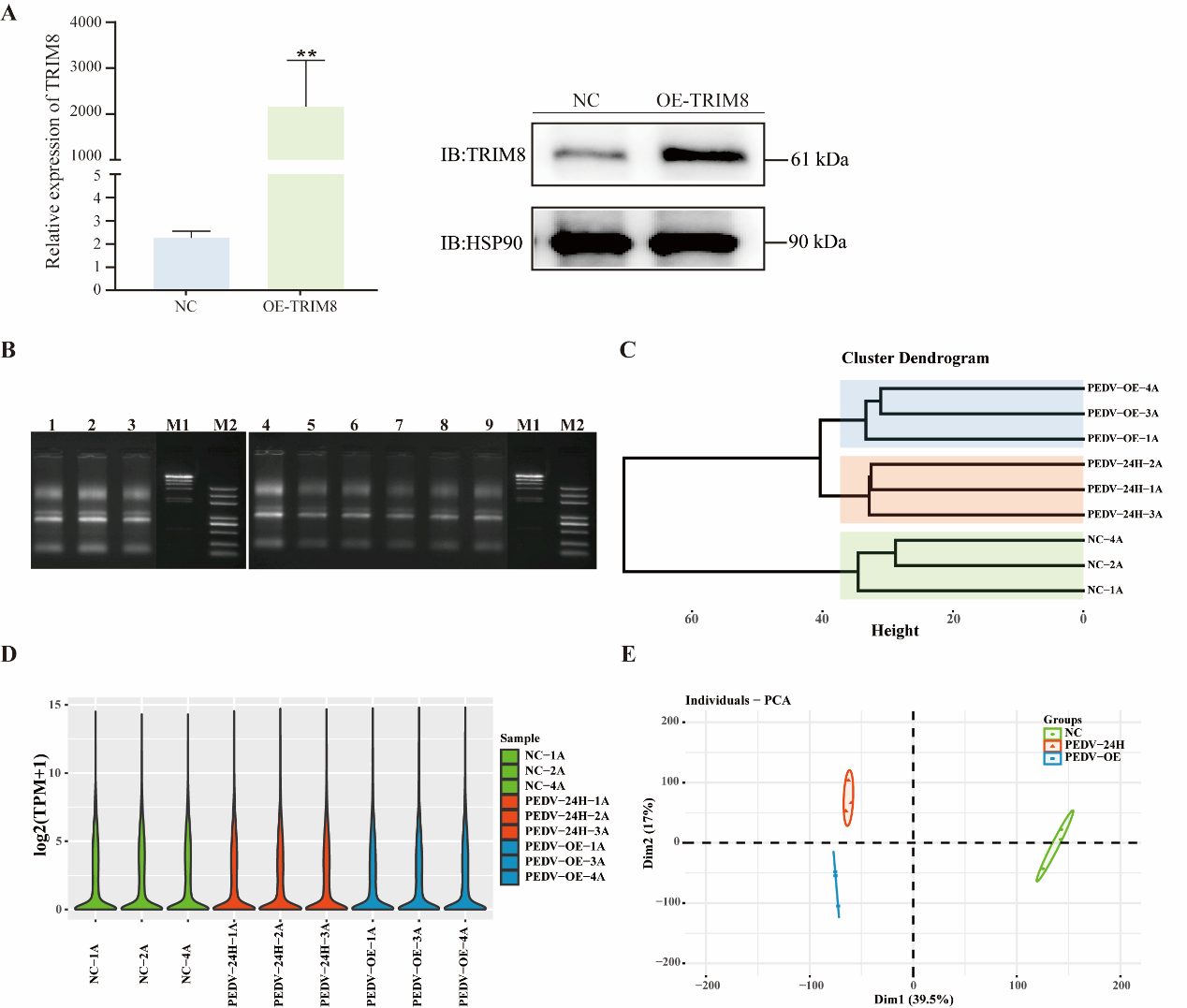


**Additional file 5.** **TRIM8 expression at mRNA (left) and protein (right) levels in TRIM8 overexpression and control cells.** OE-TRIM8: cells transfected with TRIM8 overexpression vectors; NC: cells transfected with empty vectors. ^**^*P*< 0.01.
