# Supplementary material for: TRIM8 inhibits porcine epidemic diarrhoea virus replication by targeting and ubiquitinately degrading the nucleocapsid protein
Source: Vet Res. 2025 Jan 16;56:14. doi: 10.1186/s13567-024-01443-2 (PMC11740423; doi:10.1186/s13567-024-01443-2)
Supplement: Supplementary file 6 — Additional file 6. Statistics for RNA-seq data of each sample. [file 13567_2024_1443_MOESM6_ESM.docx]

**Additional file 6. Statistics for RNA-seq data of each sample.**

| Sample ID | Clean Reads | Clean Base | Read Length | Q20 (％) | GC (％) |
| --- | --- | --- | --- | --- | --- |
| NC1 | 22097572 | 6629271600 | PE150 | 94.92 | 52.86 |
| NC2 | 22475471 | 6742641300 | PE150 | 95.03 | 53.12 |
| NC3 | 24008128 | 7202438400 | PE150 | 95.12 | 53.84 |
| P1 | 20334900 | 6100470000 | PE150 | 95.1 | 52.85 |
| P2 | 23573032 | 7071909600 | PE150 | 95.16 | 53.84 |
| P3 | 22203627 | 6661088100 | PE150 | 95.07 | 52.28 |
| P-OE1 | 24088156 | 7226446800 | PE150 | 97.08 | 52.92 |
| P-OE2 | 24053636 | 7216090800 | PE150 | 97.12 | 52.21 |
| P-OE3 | 24099237 | 7229771100 | PE150 | 97.06 | 52.73 |
