# Supplementary material for: TRIM8 inhibits porcine epidemic diarrhoea virus replication by targeting and ubiquitinately degrading the nucleocapsid protein
Source: Vet Res. 2025 Jan 16;56:14. doi: 10.1186/s13567-024-01443-2 (PMC11740423; doi:10.1186/s13567-024-01443-2)
Supplement: Supplementary file 7 — Additional file 7. Hierarchical clustering (A) and principal component (B) analyses of the samples used in RNA-seq. [file 13567_2024_1443_MOESM7_ESM.docx]

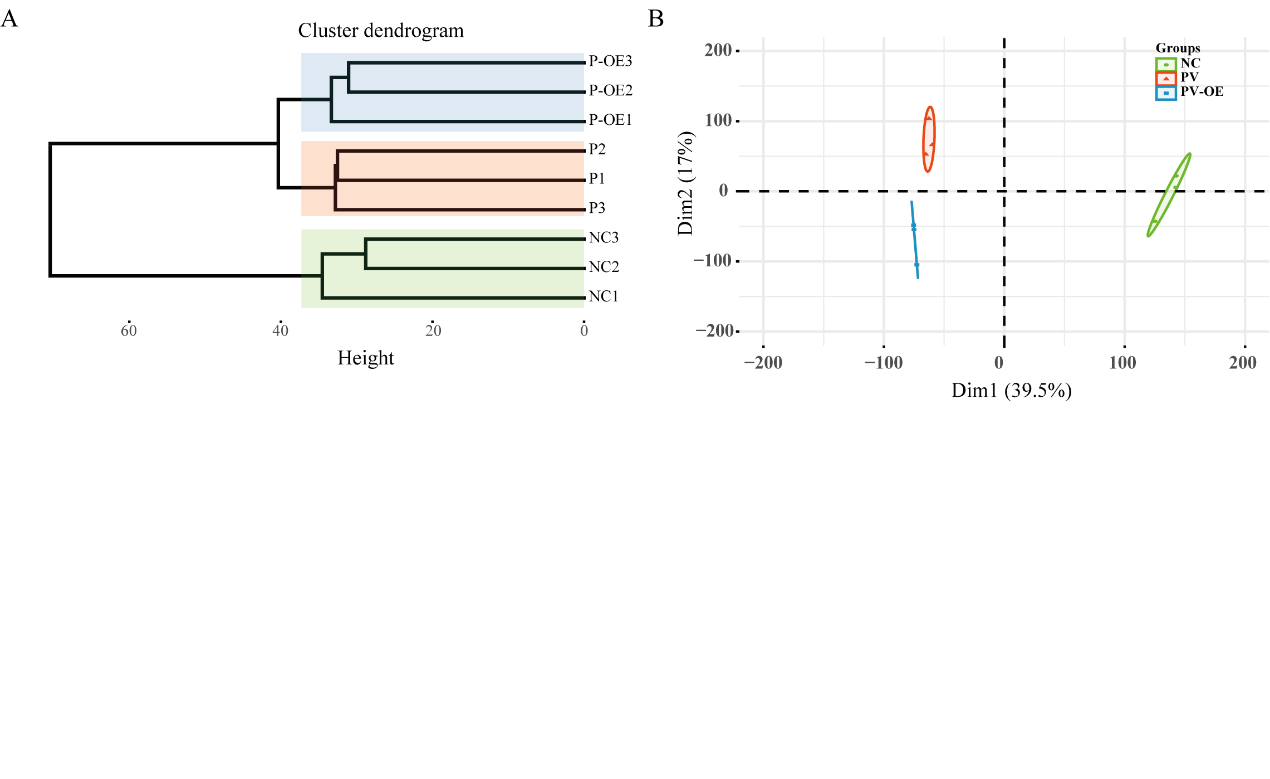


**Additional file 7.** **Hierarchical clustering (A) and principal component (B) analyses of the samples used in RNA-seq.** P-OE1, P-OE2, and P-OE3 represent PEDV-treated TRIM8 overexpression samples; P1, P2, and P3 represent PEDV-treated samples; NC1, NC2, and NC3 represent PEDV untreated samples. PV represents the PEDV-treated group; PV-OE represents the PEDV-treated TRIM8 overexpression group; NC represents the PEDV untreated group.
