# Supplementary figures and images for: TRIM8 inhibits porcine epidemic diarrhoea virus replication by targeting and ubiquitinately degrading the nucleocapsid protein
Source: Vet Res. 2025 Jan 16;56:14. doi: 10.1186/s13567-024-01443-2 (PMC11740423; doi:10.1186/s13567-024-01443-2)

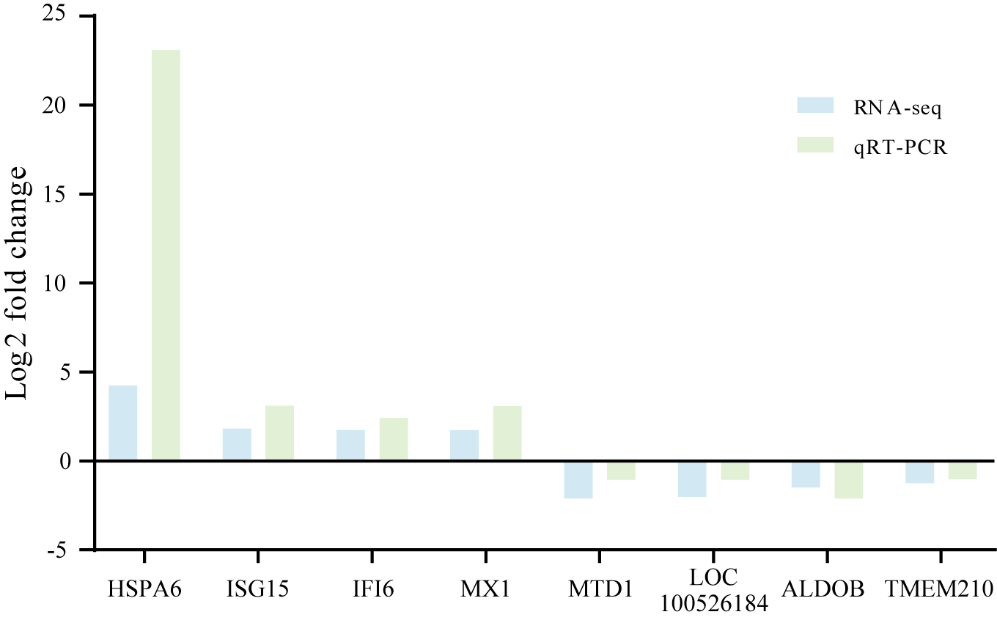


**Additional file 10. Validation of differentially expressed genes from RNA-seq data by qRT-PCR.**

Supplement: Supplementary file 10 — Additional file 10. Validation of differentially expressed genes from RNA-seq data by qRT-PCR. [file 13567_2024_1443_MOESM10_ESM.docx]
